# Predicting total body water in infants aged 0.5–24 months and the implications for measuring breast milk intake: a secondary analysis of isotope dilution data

**DOI:** 10.1016/j.ajcnut.2025.06.004

**Published:** 2025-08-14

**Authors:** Marieke AJ de Sévaux, Pernille Kæstel, Hinke Haisma, Teresa HM da Costa, Adriana Vázquez-Vázquez, Tinku Thomas, Regien Biesma, Rebecca Kuriyan, Grace Munthali, Aly Diana, Ina S Santos, Andrew P Hills, V Pujitha Wickramasinghe, Shabina Ariff, M Nishani Lucas, Caroline S Costa, Shane A Norris, Vandana Jain, Helen Mulol, Ute Feucht, Birna Thorisdottir, Inga Thorsdottir, Peter SW Davies, Cornelia U Loechl, Jonathan CK Wells

**Affiliations:** 1 Global Health Unit, Department of Health Sciences, University Medical Center Groningen, Groningen, The Netherlands; 2 Population Research Centre, Faculty of Spatial Sciences, University of Groningen, Groningen, The Netherlands; 3 Nutritional and Health-related Environmental Studies Section, Division of Human Health, Department of Nuclear Sciences and Applications, International Atomic Energy Agency, Vienna, Austria; 4 Nutrition Department, University of Brasilia, Brasilia-Federal District, Brazil; 5 Population Policy and Practice Department, University College London, Great Ormond Street Institute of Child Health, London, United Kingdom; 6 Department of Biostatistics, St John’s Medical College, Bengaluru, India; 7 Julius Global Health, University Medical Center Utrecht, The Netherlands; 8 Division of Nutrition, St. John’s Research Institute, Bengaluru, India; 9 National Institute for Scientific and Industrial Research (NISIR), Lusaka, Zambia; 10 Department of Public Health, Faculty of Medicine, Universitas Padjadjaran, Bandung, Indonesia; 11 Postgraduate Program in Epidemiology, Faculty of Medicine, Federal University of Pelotas, Pelotas, Brazil; 12 School of Health Sciences, College of Health and Medicine, University of Tasmania, Tasmania, Australia; 13 Department of Paediatrics, Faculty of Medicine, University of Colombo, Colombo, Sri Lanka; 14 Department of Pediatric and Child Health, The Aga Khan University, Pakistan; 15 SAMRC (South African Medical Research Council) Developmental Pathways for Health Research Unit, Department of Pediatrics, University of the Witwatersrand, Johannesburg, South Africa; 16 School of Human Development and Health, University of Southampton, Southampton, United Kingdom; 17 Department of Paediatrics, All India Institute of Medical Sciences, New Delhi, India; 18 Department of Paediatrics, University of Pretoria, Pretoria, South Africa; 19 Research Centre for Maternal, Fetal, Newborn, and Child Health Care Strategies, University of Pretoria, Pretoria, South Africa; 20 Maternal and Infant Health Care Strategies Unit, South African Medical Research Council, Pretoria, South Africa; 21 Faculty of Food Science and Nutrition, University of Iceland, Reykjavik, Iceland; 22 Unit of Nutrition Research, Health Science Institute, University of Iceland, Reykjavik, Iceland; 23 Department of Nutrition, Exercise and Sports, University of Copenhagen, Copenhagen, Denmark; 24 Child Health Research Centre, The University of Queensland, Brisbane, Queensland, Australia

**Keywords:** infant, total body water, body composition, anthropometry, deuterium, doubly labeled water, isotope dilution, breast milk intake

## Abstract

**Background:**

Total body water (TBW) is commonly used to derive estimates of body composition. The deuterium oxide dose-to-mother (DTM) technique for measuring breast milk intake requires an estimate of infant TBW. The DTM calculation employs a prediction equation for estimating infant TBW from body weight (TBW_pred_), but the general validity of this equation is unknown.

**Objectives:**

The objectives of this study were to assess the bias of TBW predicted by the equation used in DTM calculations (TBW_pred_); derive a new equation based on a multicountry dataset of TBW measured by isotope dilution (TBW_id_) in breastfed infants, 0.5 (2 wk) to 24 mo old; and quantify the impact of the new equation on measurements of breast milk and nonmilk water intake using the DTM technique.

**Methods:**

We conducted a secondary analysis of 3756 TBW_id_ measurements from 1457 infants, which were compared to TBW_pred_ using the Bland-Altman method and used to generate a new prediction equation. Values of breast milk intake and nonmilk water intake from 130 DTM calculation spreadsheets were compared with recalculated values using the Bland-Altman method.

**Results:**

TBW_pred_ underestimates TBW, with absolute and relative bias increasing with age (3 mo: –1.4% ± 9.2%; 24 mo: –11.3% ± 8.3%) and differential by sex (males: –6.4% ± 9.6%; females: –2.4% ± 9.9%). The best prediction equation incorporates log weight, age, and sex, explaining 93.7% of the variability in log TBW_id_. Applying this new equation in DTM calculations results in higher estimates of breast milk and nonmilk water intake; for example, in infants aged 6–12 mo, differences averaged 26 g/d (limits of agreement: –17, 69) and 13 g/d (limits of agreement: –10, 36), respectively.

**Conclusions:**

The equation used in DTM calculations provides biased TBW estimates. This has implications for measuring breast milk intake and nonmilk water intake and our understanding of nutritional needs during the first 2 y of life.

## Introduction

Total body water (TBW) refers to the total amount of water present in the body. Water is the largest component of the body and is found exclusively in the fat-free mass [1]. TBW is, therefore, widely used to derive estimates of fat-free mass as a means for measuring body composition [1]. This is particularly useful for infants and young children, where other methods are either not feasible or costly or restricted to centralized research facilities [2]. Information on infant TBW is also a requirement for estimating breast milk intake using the deuterium oxide (D_2_O) dose-to-mother (DTM) technique [3].

The most widely used protocol for calculating breast milk intake using the DTM technique, along with spreadsheets for calculations, is provided by the International Atomic Energy Agency (IAEA) [3]. This technique utilizes a 2-compartment model to assess breast milk intake [3]. Information on infant TBW is essential for the calculation of breast milk intake, but it is almost always estimated rather than directly measured. Direct measurement would involve an additional dose to the infant, increasing both the time and resources needed. Instead, the IAEA’s spreadsheets use a prediction equation to estimate infant TBW from its body weight (W) using the equation; $TBW=0.84*W^{0.82}$ [3]. This equation was derived from data from young infants in Cambridge, United Kingdom. To date, it is unclear how this equation performs in infants ≤24 mo of age or in populations from other geographic locations.

This study aimed to *1*) compare TBW predicted from weight (TBW_pred_) against measurements of TBW using isotope dilution for measurement of body composition (TBW_id_) to quantify the magnitude of bias; *2*) derive a new equation based on a multicountry dataset of TBW_id_ in breastfed infants from 0.5 to 24 mo old; and *3*) quantify the impact of the new prediction equation for TBW on measurements of breast milk and nonmilk water intake by the DTM technique.

## Methods

### Data

This study conducted a secondary analysis of data from 9 studies across 9 countries collected between 1986 and 2021 (Table 1). All included studies received approval from the relevant institutional review boards, and informed consent was provided for all participants by their mothers or parents. The majority of data were from the multicenter infant body composition reference study (MIBCRS) [4,5], and data from the other studies were included to enrich data from younger (<3 mo) and older infants (≥18 mo) and other geographic areas (Iceland, United Kingdom, and Zambia). This yielded an initial pooled dataset of 4702 TBW_id_. Records were excluded from the initial dataset when the difference between weight and the sum of fat mass and fat-free mass exceeded 0.1 kg (*n* = 202), when there was doubt about the infant’s age at measurement (*n* = 22), when isotope enrichment levels were suggestive of contamination or dosing errors (*n* = 9), or the infant was born prematurely (<37 wk) (*n* = 43).

**TABLE 1:** Characteristics and methods of data included in the pooled analysis.

Study/reference	Country	Data type	Period of data collection (years)	*n* records	Age range or approximate age at follow-up (mo)
Davies et al. [12]	United Kingdom	Cross-sectional	1986–1987	20	1.5
Haisma et al. [6,7]	Brazil	Cross-sectional	2001–2002	55	8
Jain et al. [8]	India	Cross-sectional	2012–2014	133	0.5
Multicenter infant body composition reference study [4,5]	Australia, Brazil, India, Pakistan, South Africa, and Sri Lanka	Longitudinal	2013–2019	3174^1^	3–24
Thorisdottir et al. [9]	Iceland	Cross-sectional	2014–2019	21	6
UmbiBaby [11,14,15]	South Africa	Longitudinal	2018–2020	243	1.4–24
UmbiGodisa [11,16,17]	South Africa	Cross-sectional	2018–2020	26	18
Unpublished data (study)	India and Zambia	Cross-sectional	2019–2021	45^2^	7–24
Unpublished data (follow-up of Thorisdottir et al. [9])	Iceland	Cross-sectional	2014–2019	39	12

1 Australia: 52; Brazil: 629; India: 89, Pakistan: 737; South Africa: 1136; Sri Lanka: 531.

2 India: 11; Zambia: 34.

Only breastfed infants were considered eligible for this study because the final aim was to assess the impact of a new prediction equation for TBW on breast milk and nonmilk water intake measurements by the DTM technique. Haisma et al. [6,7], Jain et al. [8], Thorisdottir et al. [9], and the unpublished data from a study in India and Zambia which was aimed at developing a DTM protocol with fewer sampling times, included only breastfed infants. The MIBCRS included mothers who intended to breastfeed exclusively until 6 mo and continue breastfeeding until ≥12 mo [4,10]. Hence, records from this sample were excluded when we could verify or had strong reason to believe that the infant was not breastfed at the time of measuring TBW, based on reported feeding practices (*n* = 670) (Supplemental Material 1). The UmbiBaby study and UmbiGodisa study were postnatal follow-up studies to the Umbiflow international study, which collected information on breastfeeding practices through interviews [11]. Only records from breastfed infants were shared for the present study. Likewise, only data from breastfed infants in the study by Davies et al. [12] and the unpublished follow-up study of Thorisdottir et al. [9] were shared for the present study, as not all infants were still breastfeeding at the time of follow-up. This resulted in a final sample size of 3756 TBW measurements in 1457 infants representing different ages (Supplemental Figure 1). For all infants, data were available on age, sex, weight, TBW_id_, and country. Length data were available for all infants except 1 from Thorisdottir et al. [9]. Although reported in Jain et al. [8], data on length from this study were not included in our analysis for practical reasons, and we do not expect their exclusion to have affected our results. Age groups were defined as follows: 0.5 mo (≤2), 3 mo (>2 to ≤4.5), 6 mo (>4.5 to ≤7.5), 9 mo (>7.5 to ≤10.5), 12 mo (>10.5 to ≤16.5), 18 mo (>16.5 to ≤20.5), and 24 mo (>20.5).

A second dataset was collated to quantify the impact of the new prediction equation for TBW on measurements of breast milk and nonmilk water intake. We had access to isotope enrichment data used for calculating these intakes using the DTM method originating from Brazil (*n* = 11) [6,7], Indonesia (*n* = 30) [13], and the studies in India (*n* = 44) and Zambia (*n* = 52) by convenience. The infants from the studies in India and Zambia were also part of the sample of TBW measurements. The aim was to create a diverse sample of male and female infants from birth to 24 mo, with variability in breast milk and nonmilk water intakes. Data from 7 records (*n* = 3 from Brazil and *n* = 4 from India) were excluded because they had unacceptable curve fitting errors (>100 mg/kg) (*n* = 2), erroneous enrichment data (*n* = 4), or incomplete available data (*n* = 1). The methods used in all studies were based on the protocol described by the IAEA [3], but the number of sampling days varied. The study in India and Zambia applied a protocol where saliva samples from mothers and infants were taken on all 14 d postadministration of deuterium to the mother. In Brazil, infants were sampled on 6 and mothers on 5-time points over 14 d, and in the Indonesian sample, a 3-sample protocol was applied over 14 d [3,4,6,7,13]. Original values of breast milk and nonmilk water intake were calculated by use of the standard Excel spreadsheet as provided on the IAEA’s website (https://www.iaea.org/resources/hhc/nutrition/breast-milk-intake/guidance-material), which applies the Cambridge equation for predicting TBW. Isotope enrichment data were subsequently transferred to a variation of the spreadsheet, which applies the equation for predicting TBW derived in the present study from the multicountry dataset of infants aged 0.5–24 mo (example available in Supplemental File 2). We then reanalyzed the “Solver” function embedded in the spreadsheet, recording the recalculated values of breast milk intake and nonmilk water intake. This resulted in a pooled dataset of 130 paired measurement points, where each infant’s TBW was calculated using both equations and the minimum breast milk intake was 53 g/d (Supplemental Figure 2). A sample size of 100 is sufficient to estimate the difference between the original and recalculated measurements of breast milk intake with an SD of 0.1 kg/d with a 20% margin of error and 95% confidence interval (CI). The data that were available were therefore deemed sufficient for our purpose.

### TBW_id_

Established protocols for measuring TBW using stable isotope dilution were applied in the individual studies, although there were some variations in the exact methodology. The protocol for measuring TBW as described by the IAEA was applied by the MIBCRS, UmbiBaby, UmbiGodisa, and the study in India and Zambia [1,4,[14], [15], [16], [17]]. In short, infants received an oral dose of D_2_O (99.8 atom% ^2^H). Saliva samples were collected before and on 1 or more occasions after administration of the dose. Jain et al. [8] also dosed the infants with D_2_O (99.9 atom% ^2^H) but took urine samples. D_2_O enrichment in saliva or urine was measured either by Fourier transform infrared spectroscopy or by isotope ratio mass spectrometry. TBW was calculated by the plateau method [18], using the weight of D_2_O consumed, the enrichment of the deuterium in the dose, and the postdose enrichment of deuterium in the saliva or urine sample, with a correction (4.1%) for nonaqueous exchange of deuterium [1].

Other studies calculated TBW using the back extrapolation method with slight variations in dosing protocols [18]. Haisma et al. [6,7] administered doubly labeled water (0.18 g/kg H_2_^18^O and 0.10 g/kg ^2^H_2_O). Davies et al. [12] administered a dose of 2.8 g/kg body weight of 10% H_2_^18^O, and the infants in the study by Thorisdottir et al. [9] received an oral dose of 0.1 g/kg body weight of >99.9 atom% ^2^H_2_O and 2.5 g/kg 10 atom% H_2_^18^O. In these studies, isotope enrichment in the pre and postdose urine samples was analyzed using isotope ratio mass spectrometry. TBW was subsequently calculated as the mean of the isotope distribution spaces of ^2^H (*V*) and ^18^O (*V*’) and corrected for nonaqueous isotope exchange as follows: $TBW=\left(\frac{V}{1.04}+\frac{V^{\prime }}{1.01}\right)/2$ [6,18]. The studies that measured TBW at multiple time points – i.e., that were longitudinal (Table 1 [[4], [5],^11^,[14], [15]]) – repeated the protocol at each measurement point. Standard quality control procedures to ensure accuracy of the data included duplicate or triplicate analyses, calibration with international reference standards, and repeat measurements when variation between replicates exceeded predefined thresholds (Fourier transform infrared spectroscopy: coefficient of variation ≥1%; isotope ratio mass spectrometry: >0.5 ‰ ^18^O or 5.0 ‰ ^2^H) [1,9,19,20]. As TBW was calculated using either the plateau or back extrapolation method, minor differences in TBW values may arise due to methodological variation [18], which we were unable to fully control for. Detailed descriptions of how TBW_id_ was measured in each individual study are provided elsewhere [1,4,[6], [7], [8], [9],^12^,[14], [15], [16], [17], [18]].

### Equation for predicting TBW_pred_

The Cambridge equation for predicting TBW_pred_ based on data from young infants is: $TBW=0.84*W^{0.82}$ [3].

### Statistical analysis

The differences between TBW_pred_ and TBW_id_ were tested using the Wilcoxon signed-rank nonparametric test for paired samples because the distribution was not normal. Spearman’s correlation coefficient was computed to quantify the association between TBW_id_ and TBW_pred_. The bias of TBW_pred_ relative to TBW_id_ was plotted and computed using the Bland-Altman method [21]. The bias was studied in the complete sample, stratified by age, sex, and country, excluding infants from the United Kingdom [12] (*n* = 20) as they were part of the sample used to develop the Cambridge TBW prediction equation. Absolute bias was calculated as ${TBW}_{pred}-{TBW}_{id}$, and relative bias as a percentage using the natural log transformation of the individual values and multiplying by 100 as follows:

$$Bias\;{TBW}_{pred}\;\left(\%\right)=\left(\ln\left({TBW}_{pred}\right)-\ln\left({TBW}_{id}\right)\right)\;x\;100\%$$

Spearman’s correlation coefficient was used to quantify the correlations between the bias of TBW_pred_ and age. To develop and cross-validate new prediction equations, the original dataset, including the infants from the United Kingdom, was randomly split into a development (80%; *n* = 3006) and a validation (20%; *n* = 750) dataset using the R-package “caret” [22]. Linear mixed models, including complete cases and a random effect for infants, taking into account the repeated TBW measurements, were used to derive new equations for predicting TBW. Nested models incorporating different combinations of the potential predictor variables infant weight (kilogram), length (centimeter), sex, and age (month) were constructed for improved accuracy. The variables TBW_id_, weight, and length were transformed and included in the models as natural log values to obtain a normal distribution. The *R*^*2*^, Akaike information criterion, and root mean square error were used to evaluate model performance, and nested models were compared using likelihood ratio tests. The coefficients of the final model were additionally compared with those of a bootstrap linear mixed regression analysis of 5000 sample replications to check the internal validity of the model. The bias of the new prediction equation compared with TBW_id_ was computed and plotted using the Bland-Altman method [21]. Spearman’s correlation coefficient was used to quantify the correlations between the bias of the new equation and weight or age. Lastly, the Bland and Altman method [21] was used to calculate the limits of agreement (LoA) of the differences between recalculated and original breast milk and nonmilk water intake. Spearman’s correlation coefficient was calculated to measure the association between the difference in original and recalculated intakes and age or mean intake. Sex differences were assessed using an independent samples t-test, and Cohen’s *d* was used as a measurement of the effect size. All analyses were performed using R statistical software (v4.2.0) [23], and *P* values <0.05 were considered statistically significant.

## Results

Characteristics of the study population are presented for the complete sample and stratified by age and country in Table 2. The female-to-male ratio was 49/51 in the complete sample and was similar in all age categories.

**TABLE 2:** Population characteristics.

	*n*	Weight^1^ (kg)	Length^1^ (cm)	Country^2^								
				Australia	Brazil	Iceland	India	Pakistan	South Africa	Sri Lanka	UK	Zambia
Overall	3756	7.9 ± 2.2	69.3 ± 8.7	52 (1.4)	684 (18)	60 (1.6)	232 (6.2)	737 (20)	1406 (37)	531 (14)	20 (0.5)	34 (0.9)
Age group^3^ (mo)												
0.5	191	3.6 ± 0.9	55.1 ± 2.2	0 (0)	0 (0)	0 (0)	132 (69)	0 (0)	39 (20)	0 (0)	20 (10)	0 (0)
3	897	6.0 ± 0.8	59.6 ± 2.5	0 (0)	172 (19)	0 (0)	0 (0)	145 (16)	443 (49)	137 (15)	0 (0)	0 (0)
6	785	7.4 ± 0.9	65.7 ± 2.6	52 (6.6)	134 (17)	21 (2.7)	89 (11)	144 (18)	226 (29)	118 (15)	0 (0)	1 (0.1)
9	593	8.4 ± 1.1	70.0 ± 2.9	0 (0)	177 (30)	0 (0)	3 (0.5)	122 (21)	176 (30)	104 (18)	0 (0)	11 (1.9)
12	617	9.3 ± 1.2	74.5 ± 3.3	0 (0)	92 (15)	39 (6.3)	7 (1.1)	114 (18)	278 (45)	76 (12)	0 (0)	11 (1.8)
18	354	10.4 ± 1.4	80.3 ± 3.6	0 (0)	63 (18)	0 (0)	1 (0.3)	106 (30)	124 (35)	50 (14)	0 (0)	10 (2.8)
24	319	11.3 ± 1.5	85.0 ± 3.5	0 (0)	46 (14)	0 (0)	0 (0)	106 (33)	120 (38)	46 (14)	0 (0)	1 (0.3)

Abbreviation: SD, standard deviation.

1 Mean ± SD.

2 *n* (%).

3 0.5 mo (≤2), 3 mo (>2 to ≤4.5), 6 mo (>4.5 to ≤7.5), 9 mo (>7.5 to ≤10.5), 12 mo (>10.5 to ≤16.5), 18 mo (>16.5 to ≤20.5), and 24 mo (>20. 5).

### Evaluation of bias by TBW_pred_

**FIGURE 1:**
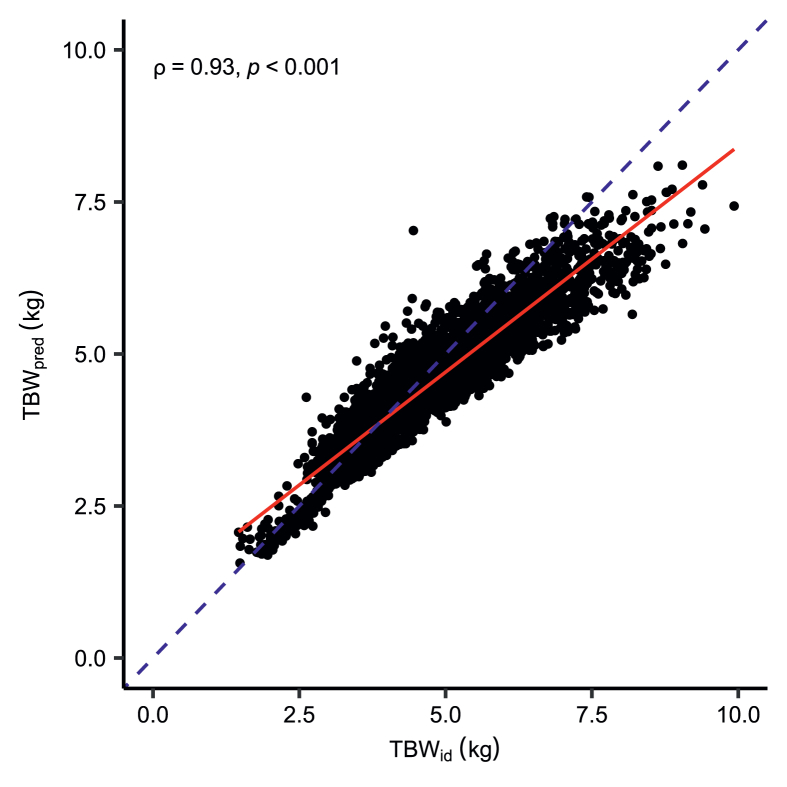
Scatterplot of total body water (TBW) as measured with isotope dilution (TBW_id_) and predicted from body weight (TBW_pred_) using the Cambridge equation (TBW_pred_ = 0.84 ∗ weight^0.82^) (*n* = 3736). The solid line (red) represents the relationship between measured and estimated TBW, and the dashed line (blue) is the identity line (y = x).

**FIGURE 2:**
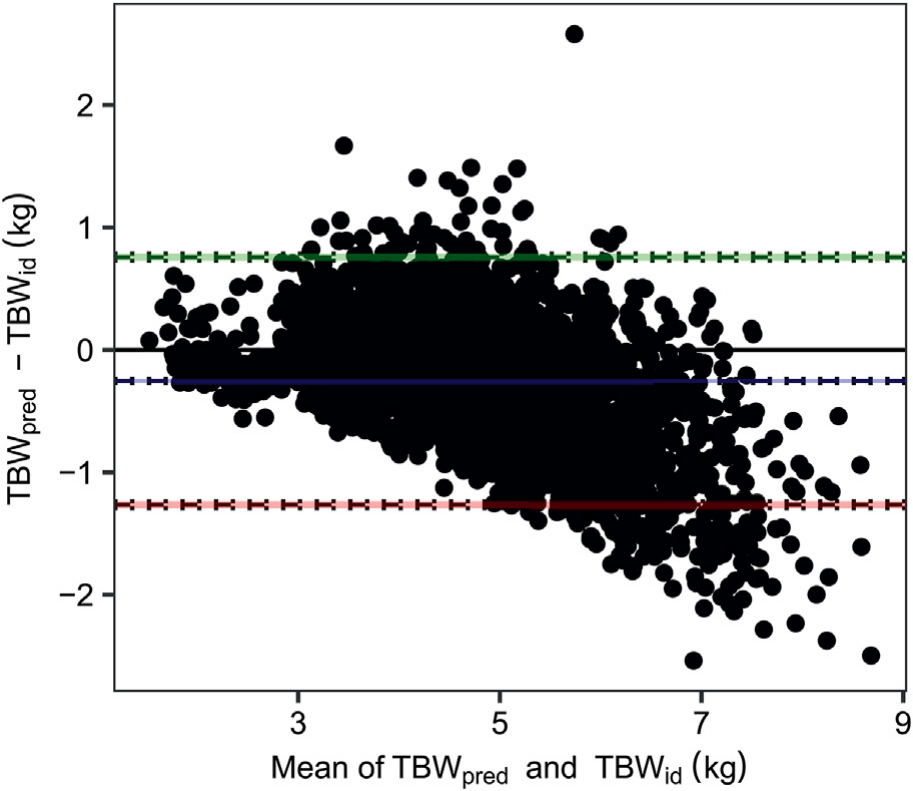
Bland-Altman plot to examine bias of total body water (TBW) predicted from body weight (TBW_pred_) using the Cambridge equation (TBW_pred_ = 0.84 ∗ weight^0.82^) relative to TBW measured with isotope dilution (TBW_id_) (*n* = 3736).

A scatterplot of TBW_id_ and TBW_pred_ is presented in Figure 1, representing the association between measured and estimated TBW. Means, SD, absolute and relative biases, and the LoA of TBW_id_ and TBW_pred_ are presented in Table 3. The mean absolute bias was –0.3 kg, and the mean relative bias was –4.4%. The Bland-Altman plot (Figure 2) illustrates that TBW_pred_ underestimated TBW, particularly in infants whose TBW was larger than 5–6 kg, as additionally shown by the statistically significant negative correlation between bias and the mean of TBW_pred_ and TBW_id_ (ρ = –0.49, *P* < 0.001). The LoA were –1.3 and 0.8 kg.

**TABLE 3:** Comparison of population means, SD, bias, and limits of agreement of total body water as measured with isotope dilution and predicted from weight.

	*n*	TBW_id_^1^ (kg)	TBW_pred_^1^ (kg)	*P* value^2^	Bias^3^ (kg)	Limits of agreement (kg)	Bias^1^^,^^4^ (%)
Overall	3736	4.83 ± 1.32	4.58 ± 1.05	<0.001	–0.25	–1.29, 0.78	–4.43 ± 9.93
Age group^5^ (mo)							
0.5	171	2.50 ± 0.59	2.35 ± 0.48	<0.001	–0.15	–0.55, 0.26	–5.49 ± 8.53
3	897	3.70 ± 0.48	3.64 ± 0.39	<0.001	–0.06	–0.74, 0.62	–1.37 ± 9.17
6	785	4.35 ± 0.55	4.32 ± 0.44	0.019	–0.03	–0.81, 0.74	–0.53 ± 8.83
9	593	5.04 ± 0.69	4.79 ± 0.51	<0.001	–0.25	–1.22, 0.72	–4.71 ± 9.77
12	617	5.56 ± 0.74	5.22 ± 0.56	<0.001	–0.35	–1.39, 0.70	–6.09 ± 9.67
18	354	6.38 ± 0.86	5.71 ± 0.61	<0.001	–0.67	–1.88, 0.54	–10.76 ± 9.51
24	319	6.91 ± 0.92	6.15 ± 0.65	<0.001	–0.76	–1.92, 0.41	–11.30 ± 8.34
Sex							
Female	1824	4.64 ± 1.26	4.49 ± 1.03	<0.001	–0.15	–1.12, 0.82	–2.35 ± 9.89
Male	1912	5.01 ± 1.35	4.66 ± 1.07	<0.001	–0.35	–1.40, 0.69	–6.41 ± 9.56
Country							
Australia	52	4.29 ± 0.47	4.26 ± 0.33	0.613	–0.04	–0.73, 0.65	–0.54 ± 8.09
Brazil	684	5.12 ± 1.24	4.75 ± 0.94	<0.001	–0.37	–1.33, 0.59	–6.62 ± 8.53
Iceland	60	5.27 ± 0.88	5.20 ± 0.58	0.287	–0.07	–1.03, 0.89	–0.44 ± 9.39
India	232	3.15 ± 1.15	3.06 ± 1.13	<0.001	–0.08	–0.69, 0.53	–2.80 ± 9.30
Pakistan	737	5.16 ± 1.30	4.72 ± 0.97	<0.001	–0.44	–1.41, 0.54	–7.81 ± 8.49
South Africa	1406	4.74 ± 1.23	4.70 ± 1.03	0.077	–0.04	–0.97, 0.89	–0.06 ± 9.40
Sri Lanka	531	4.94 ± 1.26	4.42 ± 0.85	<0.001	–0.52	–1.64, 0.60	–9.80 ± 10.19
Zambia	34	5.48 ± 0.88	5.05 ± 0.67	<0.001	–0.43	–1.21, 0.35	–7.70 ± 6.78

Abbreviations: SD, standard deviation; TBW, total body water; TBW_id_, total body water measured with isotope dilution; TBW_pred_, total body water predicted from weight.

1 Mean ± SD.

2 Wilcoxon signed-rank nonparametric test for paired samples.

3 Bias (kg) = TBW_pred_ – TBW_id_.

4 Bias (%) = $\left(\ln\left({TBW}_{pred}\right)-\ln\left({TBW}_{id}\right)\right)\;x\;100\%$.

5 0.5 mo (≤2), 3 mo (>2 to ≤4.5), 6 mo (>4.5 to ≤7.5), 9 mo (>7.5 to ≤10.5), 12 mo (>10.5 to ≤16.5), 18 mo (>16.5 to ≤20.5), 24 mo (>20. 5).

Beyond the age of ∼6 mo, the underestimation of TBW by TBW_pred_ increased with age, as demonstrated by the statistically significant negative correlation between bias and age (ρ = –0.40, *P* < 0.001). Bland-Altman plots per age category are shown in Supplemental Figure 3. The underestimation of TBW by TBW_pred_ was significantly greater in male infants compared to female infants, with a mean difference of 0.2 kg [95% CI: 0.17, 0.24 kg; *t*(3731) = 12.47, *P* < 0.001)]. Supplemental Figure 4 presents Bland-Altman plots by sex. Additionally, the mean bias varied by country, with TBW_pred_ underestimating TBW by >6.6% in Brazil, Pakistan, Sri Lanka, and Zambia, whereas the negative bias was <0.6% in Australia, Iceland, and South Africa. Country-specific Bland-Altman plots are shown in Supplemental Figure 5.

### New predictive equations

The development and validation samples had comparable distributions of age, sex, weight, length, and country of origin. New equations predicting lnTBW_id_ in the development sample (*n* = 3006) are presented in Table 4. The natural logarithm of weight alone accounted for 92.8% of the variability in lnTBW_id_ (model 1). Including age resulted in 93.6% of the variability explained by the model (model 2), with a significant improvement in model fit from a likelihood ratio test [*X*^2^(1) = 388, *P* < 0.001]. Including sex increased the explained variability by another 0.1% (model 4), with a significant improvement in model fit compared with model 2 [*X*^2^(1) = 104, *P* < 0.001]. Including length in the model introduced multicollinearity indicated by high variance inflation factors (>16), and it was therefore excluded. Replacing weight with length in the models resulted in poorer performance compared with models that included weight (results not shown). Model 4 was, therefore, considered the most favorable. Bootstrapping resulted in very similar regression coefficients (Table 4). Converting the equation from the natural logarithmic scale back to its original scale results in the following prediction equation: TBW =0.951 ∗ weight^0.729^ ∗ 1.009^age^ ∗ 1.044^(female = 0; male = 1)^, where weight is measured in kilogram and age in month.

**TABLE 4:** Linear mixed models for predicting the natural logarithm of total body water in the development sample [*n* = 3006 data points from 9 countries (Australia, Brazil, Iceland, India, Pakistan, South Africa, Sri Lanka, United Kingdom, and Zambia)] with a random effect for infants to account for repeated measurements.

Model	Predictor	Coefficient	SE of coefficient	Bootstrapped coefficient^1^	Bootstrapped SE^1^	AIC	R^2^	RMSE
1	Constant	–0.288	0.012	–0.288	0.012	-5750.7	0.928	0.07
	lnWeight	0.895	0.006	0.896	0.006			
2	Constant	–0.046	0.016	–0.046	0.018	-6136.8	0.936	0.06
	lnWeight	0.740	0.009	0.740	0.009			
	Age	0.008	0.000	0.008	0.000			
3	Constant	–0.298	0.012	–0.298	0.012	-5806.5	0.928	0.07
	lnWeight	0.892	0.006	0.892	0.006			
	Sex (if male)	0.035	0.004	0.035	0.004			
4	Constant	–0.050	0.016	–0.050	0.016	-6239.0	0.937	0.06
	lnWeight	0.729	0.009	0.729	0.009			
	Age	0.009	0.000	0.009	0.000			
	Sex (if male)	0.043	0.004	0.042	0.004			

Abbreviations: AIC, Akaike Information Criterion; ln, natural logarithm; RMSE, root mean square error; SE, standard error; TBW_id_, total body water measured with isotope dilution.

1 Results from a bootstrap linear mixed regression analysis of 5000 sample replications

### Cross-validation of the new equation

The mean TBW_id_ in the validation sample (*n* = 750) was 4.79 kg (SD = 1.29), and the mean TBW estimated by the new equation based on weight, age, and sex (newTBW_pred_) was 4.80 kg (SD = 1.25). A Wilcoxon signed-rank test indicated that the difference between TBW_id_ and newTBW_pred_ was not significant (*P* = 0.94). The Spearman correlation coefficient between TBW_id_ and newTBW_pred_ was 0.95 (*P* < 0.001).

**FIGURE 3:**
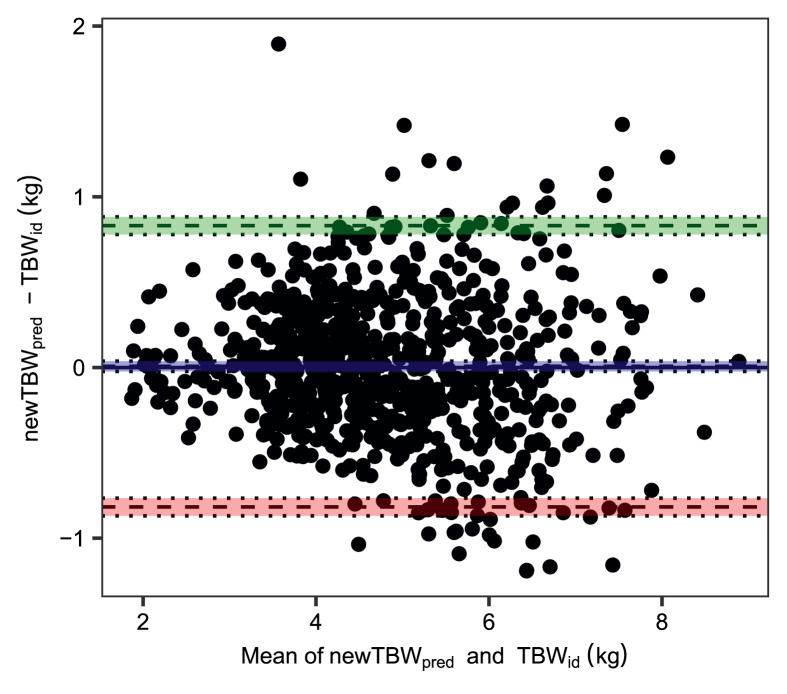
Bland-Altman plot of total body water (TBW) measured with isotope dilution (TBW_id_) and predicted by the generated equation estimating TBW based on infant weight (kilogram), age (month), and sex [newTBW_pred_ = 0.951 ∗ weight^0.729^ ∗ 1.009^age^ ∗ 1.044^(female = 0; male = 1)^].

**FIGURE 4:**
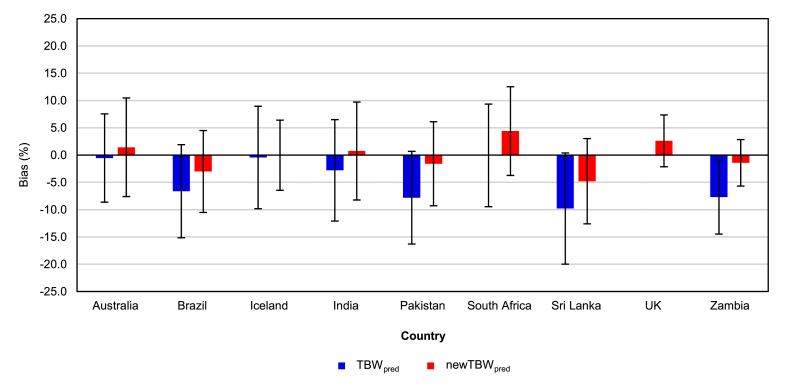
Mean relative bias (±SD) of total body water (TBW) predicted from body weight (TBW_pred_) using the Cambridge equation (TBW_pred_ = 0.84 ∗ weight^0.82^) in the complete dataset [excluding the United Kingdom (UK)] (*n* = 3736) and TBW predicted from body weight (kilogram), age (month), and sex [newTBW_pred_ = 0.951 ∗ weight^0.729^ ∗ 1.009^age^ ∗ 1.044^(female = 0; male = 1)^] in the validation dataset (20% of complete dataset; *n* = 750). Relative bias was calculated as $\left(\ln\left({newTBW}_{pred}\right)-\ln\left({TBW}_{id}\right)\right)\;x\;100\%$. ln, natural logarithm; SD, standard deviation.

The mean absolute bias of newTBW_pred_ was 0.01 kg (LoA: –0.83, 0.85), and the mean relative bias was 0.35%. The Bland-Altman plot (Figure 3) illustrates that bias was randomly distributed around 0. The bias was not correlated with weight (ρ = 0.06, *P* = 0.13) or age (ρ = 0.03, *P* = 0.41). The mean bias of females and males was similar (*P* = 0.33), with bias averaging 0.02 kg (SD = 0.40) for females and –0.01 kg (SD = 0.44) for males. A comparison of country-related bias of TBW_pred_ in the complete sample (excluding the United Kingdom) and newTBW_pred_ in the validation sample is presented in Figure 4, illustrating that the tendency toward a negative bias observed for TBW_pred_ was not present for newTBW_pred_ in any of the countries.

### Implications for measurements of breast milk and nonmilk water intake

The 130 spreadsheets used to recalculate breast milk and nonmilk water intake originated from infants between the ages of 1 and 24 mo (mean = 10.3, SD = 5.4), with 73 (56%) of them being female. The mean weight at the time of administering the dose of deuterium was 7.9 kg (range = 3.2–12.7, SD = 1.6). Before recalculation, the mean breast milk intake was 701 g/d (SD = 237), and the mean nonmilk water intake was 294 g/d (SD = 234).

**FIGURE 5:**
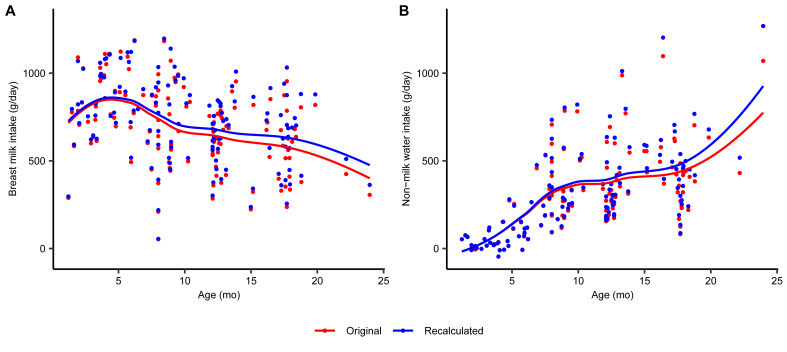
Scatterplot including loess curves of original and recalculated estimates of (A) breast milk intake (gram/day) and (B) nonmilk water intake (gram/day) by infant age (month).

Recalculating breast milk and nonmilk water intake using the newly derived equation for estimating newTBW_pred_ resulted in overall higher estimates of both breast milk and nonmilk water intake (Figure 5). The difference between recalculated and original breast milk intake ranged from –36 to 89 g/d, with a mean of 31 g/d (LoA: –18, 81). In relative terms, calculating the bias as $\left(\frac{recalculated-original}{\left(recalculated+original\right)/2}*100\%\right)$, the mean difference between original and recalculated values of breast milk intake was 4.9% (range: –3.3–18.4). For nonmilk water intake, the difference between recalculated and original values ranged from –10 to 199 g/d, with a mean of 19 g/d (LoA: –31, 68), which equates to 6.4% (range: –200–422) in relative terms.

**FIGURE 6:**
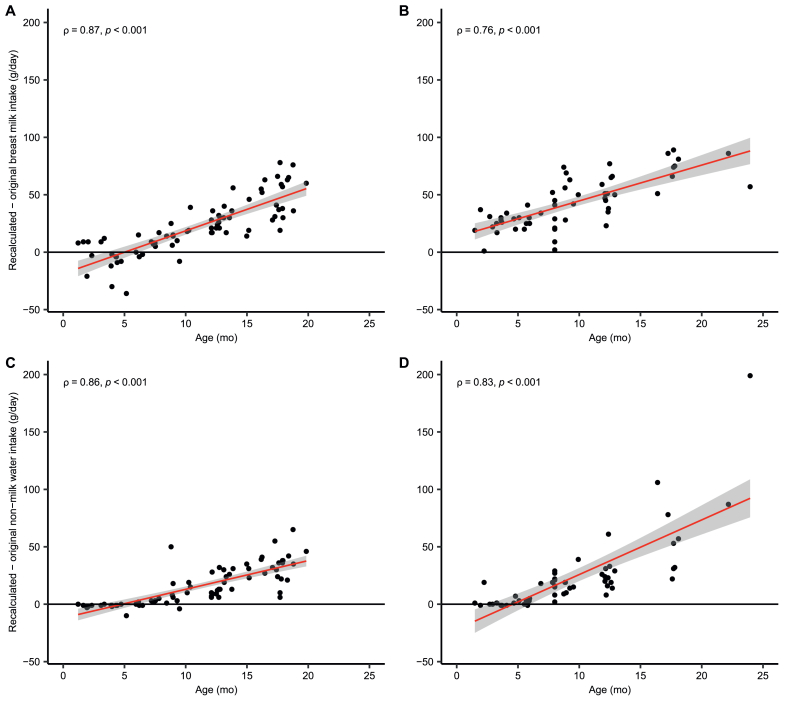
Scatterplots of the difference between recalculated and original breast milk intake (gram/day) and age (month) in (A) female (*n* = 73) and (B) male (*n* = 57) infants, and the difference between recalculated and original nonmilk water intake (gram/day) and age in (C) female and (D) male infants. The red line represents the relationship between the differences and age.

Age was strongly correlated with the difference between original and recalculated values of breast milk intake (ρ = 0.64, *P* < 0.001) and nonmilk water intake (ρ = 0.82, *P* < 0.001) (Figure 6). The mean difference between recalculated and original breast milk intake was 26 g/d (LoA: –17, 69) for infants aged 6–12 mo and 46 g/d (LoA: 5, 87) for infants aged 12 mo or older. The mean difference between recalculated and original nonmilk water intake was 13 g/d (LoA: –10, 36) for infants aged 6–12 mo and 33 g/d (LoA: –25, 91) for infants aged 12 mo or older. In addition, the differences between the 2 values increased in absolute terms with higher values of intake in infants older than ∼6 mo. Mean breast milk intake for infants >6 mo $\left(\frac{original+recalculated}{2}\right)$ was positively correlated with the difference between recalculated and original (recalculated – original) intake (ρ = 0.28, *P* = 0.01). A strong positive correlation was also observed in the case of nonmilk water intake (ρ = 0.70, *P* < 0.001). Furthermore, the differences between recalculated and original intakes were greater for males than for females (Figure 6). The mean recalculated breast milk intake was 43 g/d (LoA: –0.1, 86) greater than the original intake in males and 22 g/d (LoA: –25, 70) greater in females. A significant sex difference was observed (*P* < 0.001), with a strong effect size indicated by Cohen’s *d* (*d* = –0.87; 95% CI: –1.24, –0.51). Notably, recalculated breast milk intake for females younger than 6–7 mo was lower than the original value for a number of infants (Figure 6), with the mean difference being –3.8 g/d (–0.12%) for females younger than 7 mo. For nonmilk water intake, the mean difference between the recalculated and original values was 23 g/d (LoA: –41, 87) for males and 16 g/d (LoA: –17, 49) for females. This sex difference was not significant (*P* = 0.12), with a small to medium effect size indicated by Cohen’s *d* (*d* = –0.30; 95% CI: –0.64, 0.05).

## Discussion

This study quantified the bias of an equation for estimating TBW in breastfed infants, widely used in spreadsheets to calculate breast milk intake, relative to TBW_id_ in a large multicountry sample spanning a wide age range. Our main findings were that the old equation increasingly underestimates TBW in older infants, with the bias greater for males than for females. Second, we generated a new predictive equation for newTBW_pred_ [TBW=0.951 ∗ weight^0.729^ ∗ 1.009^age^ ∗ 1.044^(female = 0; male = 1)^], which predicted TBW with a mean error of 0.35% in the validation sample. We finally showed that applying this equation in the calculations for breast milk intake and nonmilk water intake using the DTM technique leads to overall higher estimated values, particularly for older and male infants.

The difference in TBW between the Cambridge equation and isotope dilution became increasingly greater with higher TBW values and thus with age. Given that all infants in the present study were breastfed, the observed underestimation of TBW beyond ∼6 mo is unlikely to stem from differences in body composition related to feeding type [24]. More plausibly, it reflects the changing association by age between weight, fat, and lean mass [4,25]. In infants under 3 mo, fat comprises a substantial portion of infant weight, but fat accumulation slows rapidly as infants get older [4,25]. Thus, heavier 3-mo-olds would show smaller increases in predicted TBW compared to older infants. Because the Cambridge equation was derived from young infants, it likely persists in the assumption that a higher weight corresponds predominantly to fat, leading to the observed underestimation of TBW in older infants.

Studies show that males have higher levels of fat-free mass, although fat is similar between sexes [4,25]. An equation that over-attributes greater weight to greater fat mass helps explain why the underestimation of TBW in our study was more than twice as great for males than for females.

The Cambridge equation for TBW estimation showed varying degrees of bias in the countries represented in our dataset, ranging from an underestimation of almost 10% in the Sri Lankan sample to 0.06% in the sample from South Africa. We showed that the new prediction equation reduced the negative bias observed across various countries. Notably, countries with near-0 bias using the Cambridge equation (Australia, Iceland, India, and South Africa) exhibit a slightly positive bias with the new equation. This likely stems from the Cambridge equation’s overall negative bias. By addressing this issue, the new equation achieves a more balanced distribution of biases, resulting in both positive and negative values that are not significant in any country. It should additionally be noted that although the evidence remains sparse, previous studies have described differences in body composition during infancy and early childhood related to ancestry [[26], [27], [28]]. For instance, infants of South Asian ancestry have less fat-free mass than infants of White European origin, even when correcting for size [27]. Although the Cambridge sample was mainly of European ancestry, interpreting our findings or hypothesizing about the underlying mechanism of the observed country differences in the present study is difficult without sufficient knowledge about the ancestry of our sample. Nevertheless, a key strength of this study was its multicountry sample, representing diverse geographic and economic contexts. This emphasizes the need to shed further light on regional differences in body composition during the first 2 y of life and to develop prediction equations tailored to specific countries or populations with shared ancestry to enhance the accuracy of TBW estimates.

Another strength of this analysis was that TBW_id_ is a reference method for measuring body composition [2]. Our study population was substantially larger than prior studies on TBW prediction equations, and the only one focused on infants [[29], [30], [31], [32]]. Cross-validation of the generated equation estimating newTBW_pred_ showed a mean bias of 0.35%, with no correlation to age, weight, or sex. Bootstrapping supported the robustness and reliability of the equation, as the estimates produced were nearly identical to the original model. This indicates that this equation resolves the majority of the systematic bias in the Cambridge equation and predicts TBW more accurately for infants ≤24 mo of age. Additional out-of-sample validation in diverse populations could further strengthen the robustness and generalizability of the new equation.

Our findings have important implications for estimating breast milk and nonmilk water intake using the DTM technique. Replacing the original Cambridge equation with the newly derived TBW equation resulted in higher estimates of both breast milk intake and nonmilk water intake for infants older than ∼6 mo. It was reassuring that the mean difference between the recalculated and original values was only 4.9% for breast milk intake and 6.4% for nonmilk water intake, although these differences increased with age. As anticipated, the age- and sex-related patterns observed in the original TBW equation reappeared for fluid intakes, reflecting their integration into the new equation. These findings imply that there is some error in previous reports of fluid intakes based on the DTM technique, particularly for young females and older infants. Future studies should be aware of this and should consider adopting the equation generated in the present study. This equation could also be applied in research with only basic anthropometric data or in clinical practice to guide dialysis or medication prescriptions [29,33]. However, the new equation was generated from a sample of breastfed infants, and its validity needs to be evaluated for nonbreastfed infants. Although intake recalculations were based on studies with varying protocols, this is not expected to influence the difference between the original and recalculated values of breast milk or nonmilk water intake.

This study also had some limitations. First, although our study population originated from various countries, they may not represent the general population. In addition, the ages at which TBW was measured differed between countries. Second, the generated equations for estimating TBW have residual error and R^2^ values of 93%–94% (Table 4). Although it is not possible to resolve all errors, we strove to address them to the best of our ability. Third, we had a limited number of spreadsheets to recalculate intake in infants aged >18 mo and a few <3 mo. The smaller sample sizes for these ages may have led to an underestimation of the effect of the new prediction equation for TBW on estimates of breast milk and nonmilk water intake in older infants and female infants younger than ∼7 mo. Finally, it is important to acknowledge the plausible slight overestimation of TBW in our data. The majority of the TBW measurements in this study, originating from the MIBCRS, UmbiBaby, UmbiGodisa, and the studies in India and Zambia, were calculated using the plateau method, which has been reported to overestimate TBW by 7%–8% [18]. However, several differences in the protocols of the included studies likely reduced this bias: postdose samples were collected after 2 or 3 h instead of the 5 h in the study by Davies and Wells [18], measurements were taken in saliva rather than urine, and breastfeeding was restricted to within 1 h postdose in the MIBCRS. These factors limit the loss of isotope from the body and fluid intake during the equilibration period, thereby reducing the bias [1]. Although we cannot quantify the exact magnitude of overestimation, it is unlikely to exceed more than a few percentage points, which would then also be present in the recalculated intake values.

In conclusion, the Cambridge equation for predicting TBW, applied in the DTM method for calculating breast milk intake, increasingly underestimates TBW with age, shows greater bias in males, and varies in accuracy across countries. Our newly generated equation improved the estimation of TBW and resulted in higher breast milk and nonmilk water intakes for older infants and lower breast milk intake estimates in females under ∼7 mo. This has important implications for understanding nutritional needs during the first 2 y of life and could also be applied in clinical practice when pharmacologic or dialysis dosages are tailored to anthropometry.

## Author contributions

The authors’ responsibilities were as follows – MAJdS, JCKW, PK, HH: designed research; HH, TT, RK, GM, AD, ISS, APH, VPW, SA, MNL, CSC, SAN, VJ, HM, UF, BT, IT, PSWD, JCKW: provided essential data; MAJdS: analyzed data and wrote the paper; JCKW: had primary responsibility for final content; PK, HH, RB, THMdC, AV-V, TT, CUL, JCKW: provided critical feedback on the interpretation of results and manuscript drafts; and all authors: read and approved the final manuscript.

## Data availability

Data described in the manuscript, code book, and analytic code may be made available upon request pending application to the corresponding author and approval by the co-authors involved and only for the purpose of validating the results presented in this study. The multicenter infant body composition reference study resources, however, are publicly and freely available at https://doi.org/10.17605/OSF.IO/EUXR2.

## Funding

MAJdS received a PhD grant from the University Medical Center Groningen. The funder had no involvement or restrictions regarding this publication.

## Conflict of interest

The authors report no conflicts of interest.

## References

[1] IAEA (2009).

[2] Demerath E.W., Fields D.A. (2014). Body composition assessment in the infant. Am. J Hum. Biol..

[3] IAEA (2010).

[4] Murphy-Alford A.J., Johnson W., Nyati L.H., Santos I.S., Hills A.P., Ariff S. (2023). Body composition reference charts for infants from birth to 24 months: multicenter Infant Body Composition Reference Study. Am. J Clin. Nutr..

[5] Kuriyan R., Hills A.P., Murphy-Alford A., Padmanabha R., Nyati L.H., Byrne N.M. (2024). Body composition of infants at 6 months of age using a 3-compartment model. Eur. J Clin. Nutr..

[6] Haisma H., Coward W.A., Visser G.H., Vonk R., Wells J.C., Wright A. (2006). Socio-economic and environmental factors influence energy utilization in Brazilian breast-fed infants. J Nutr.

[7] Haisma H., Wells J.C., Coward W.A., Filho D.D., Victora C.G., Vonk R.J. (2005). Complementary feeding with Cow’s milk alters sleeping metabolic rate in breast-fed infants. J Nutr.

[8] Jain V., Kurpad A.V., Kumar B., Devi S., Sreenivas V., Paul V.K. (2016). Body composition of term healthy Indian newborns. Eur. J Clin. Nutr..

[9] Thorisdottir B., Odinsdottir T., Gunnlaugsson G., Eaton S., Fewtrell M.S., Vázquez-Vázquez A. (2023). Metabolizable energy content of breastmilk supports normal growth in exclusively breastfed Icelandic infants to age 6 months. Am. J Clin. Nutr..

[10] Santos I.S., Costa C.S., Hills A.P., Ariff S., Wickramasinghe V.P., Norris S. (2024). Infant body composition at 6 and 24 months: what are the driving factors?. Eur. J Clin. Nutr..

[11] Vannevel V., Vogel J.P., Pattinson R.C., Adanu R., Charantimath U., Goudar S.S. (2022). Antenatal Doppler screening for fetuses at risk of adverse outcomes: a multicountry cohort study of the prevalence of abnormal resistance index in low-risk pregnant women. BMJ Open.

[12] Davies P.S., Wells J.C., Lucas A. (1994). Adjusting milk intake for body size in early infancy. Early Hum. Dev..

[13] Gibson R.S., Rahmannia S., Diana A., Leong C., Haszard J.J., Hampel D. (2020). Association of maternal diet, micronutrient status, and milk volume with milk micronutrient concentrations in Indonesian mothers at 2 and 5 months postpartum. Am. J Clin. Nutr..

[14] Nel S., Feucht U.D., Mulol H., Wenhold F.A. (2023). Association of prenatal Placental Function with Anthropometry and Body Composition through 2 years of Age in South African Infants: the UmbiBaby Study. J Nutr.

[15] Feucht U., Mulol H., Vannevel V., Pattinson R. (2021). The ability of continuous-wave Doppler ultrasound to detect fetal growth restriction. PLOS One.

[16] Nyofane M., Hoffman M., Mulol M., Botha T., Vannevel V., Pattinson R. (2022). Early childhood growth parameters in South African children with exposure to maternal HIV infection and placental insufficiency. Viruses.

[17] Mulol H., Nel S., Wenhold F.A., Feucht U.D. (2025). Exploring infant size and body composition at 18 months: an ambidirectional peri-urban South African cohort study. Matern. Child Nutr..

[18] Davies P.S., Wells J.C. (1994). Calculation of total body water in infancy. Eur. J Clin. Nutr..

[19] IAEA (2011).

[20] IAEA (2011).

[21] Bland J.M., Altman D.G. (1986). Statistical methods for assessing agreement between two methods of clinical measurement. Lancet.

[22] Kuhn M. (2008). Building predictive models in R using the caret package. J Stat. Softw..

[23] R Core Team (2022).

[24] Gale C., Logan K.M., Santhakumaran S., Parkinson J.R., Hyde M.J., Modi N. (2012). Effect of breastfeeding compared with formula feeding on infant body composition: a systematic review and meta-analysis. Am. J Clin. Nutr..

[25] Wells J.C., Davies P.S., Fewtrell M.S., Cole T.J. (2020). Body composition reference charts for UK infants and children aged 6 weeks to 5 years based on measurement of total body water by isotope dilution. Eur. J Clin. Nutr..

[26] Alexander T., Conlon C.A., Gamble G., von Hurst P.R., van Dorp L., Ichhpuniani B. (2020). Body composition of New Zealand-born term babies differs by ethnicity, gestational age and sex. Early Hum. Dev..

[27] Stanfield K.M., Wells J.C., Fewtrell M.S., Frost C., Leon D.A. (2012). Differences in body composition between infants of South Asian and European ancestry: the London Mother and Baby Study. Int. J Epidemiol.

[28] Wiechers C., Kirchhof S., Maas C., Poets C.F., Franz A.R. (2019). Neonatal body composition by air displacement plethysmography in healthy term singletons: a systematic review. BMC Pediatr.

[29] Wells J.C., Fewtrell M.S., Davies P.S., Williams J.E., Coward W.A., Cole T.J. (2005). Prediction of total body water in infants and children. Arch. Dis. Child..

[30] Friis-Hansen B. (1957). Changes in body water compartments during growth. Acta. Paediatr. Suppl. (Upps).

[31] Mellits E.D., Cheek D.B. (1970). The assessment of body water and fatness from infancy to adulthood. Monogr. Soc. Res. Child Dev..

[32] Morgenstern B.Z., Mahoney D.W., Warady B.A. (2002). Estimating total body water in children on the basis of height and weight: a reevaluation of the formulas of Mellits and Cheek. J Am. Soc. Nephrol..

[33] NKF-DOQI clinical practice guidelines for peritoneal dialysis adequacy (1997). National Kidney Foundation, Am. J Kidney Dis..

