# Supplementary material for: Predicting total body water in infants aged 0.5–24 months and the implications for measuring breast milk intake: a secondary analysis of isotope dilution data
Source: Am J Clin Nutr. 2025 Aug 14;122(3):820–9. doi: 10.1016/j.ajcnut.2025.06.004 (PMC12489350; doi:10.1016/j.ajcnut.2025.06.004)
Supplement: Supplementary file 1 [file mmc1.docx]

**Supplementary Material 1:** Data availability of variables related to reported feeding practices by country.

The tables in this file provide an overview of information available on reported feeding practices and the number of excluded records from the Multicenter Infant Body Composition Reference Study (MIBCRS) sample (1, 2). The initial MIBCRS sample consisted of 4,077 records, of which 3,844 records remained after applying the first set of exclusion criteria. Records from the remaining sample were excluded when we could verify or had strong belief that the infant was not breastfed at the respective measurement point. There was no information on reported feeding practices available from Australia, India, and a subset of the infants from South Africa (*n* rows = 219). Age groups were defined as follows: 3 mo (>2 – ≤4.5), 6 mo (>4.5 – ≤7.5), 9 mo (>7.5 – ≤10.5), 12 mo (>10.5 – ≤16.5), 18 mo (>16.5 – ≤20.5), 24 mo (> 20. 5)

**Brazil**

| Age group | *n* | ‘Are you currently breastfeeding?’^1^ | ‘How often do you give breast milk?’^2^ | ‘How often do you give formula?’^3^ | Freq. of giving breast milk in the last 28 days = ‘never’ | Freq. of giving breast milk in the last 28 days = ‘1-3 times a week’ & currently breastfeeding = ‘no’ | Freq. of giving breast milk last in the 28 days = NA^4^ & Freq. of formula feeding last 28 days = ‘>3x a day’ | Freq. of giving breast milk in the last 28 days = NA^4^ & Freq. of formula feeding last 28 days = ‘1-3x a day’ | Record of the same infant at 3 or 6 mo was excluded |
| --- | --- | --- | --- | --- | --- | --- | --- | --- | --- |
|  |  | *n* available records | | | *n* excluded records | | | | |
| 3 | 194 | 186 | 194 | 193 | 21 | 1 | 0 | 0 | - |
| 6 | 175 | 163 | 175 | 175 | 40 | 0 | 0 | 0 | 0 |
| 9 | 186 | 165 | 186 | 186 | 64 | 0 | 0 | 0 | 0 |
| 12 | 166 | 136 | 166 | 166 | 74 | 0 | 0 | 0 | 0 |
| 18 | 170 | 124 | 170 | 170 | 107 | 0 | 0 | 0 | 0 |
| 24 | 164 | 129 | 164 | 163 | 118 | 0 | 0 | 0 | 0 |
| Total | 1055 | 903 | 1055 | 1053 | 424 | 1 | 0 | 0 | 0 |

^1’^Yes’ or ‘No’

^2^’>3 times a day’ or ‘1 – 3 times a day’ or ‘>3 times a week’ or ‘1 – 3 times a week’ or ‘Never’

^3^>3 times a day’ or ‘1 – 3 times a day’ or ‘1 – 3 times a month’ or ‘>3 times a week’ or ‘1 – 3 times a week’ or ‘Never’

^4^NA = missing value

**Pakistan**

| **Age group** | **n** | **‘Are you currently breastfeeding?’^1^** | **‘How often do you give breast milk?’^2^** | **‘How often do you give formula?’^3^** | **Freq. of giving breast milk in the last 28 days = ‘never’** | **Freq. of giving breast milk last in the 28 days = NA^4^ & Freq. of formula feeding last 28 days = ‘>3x a day’** | **Freq. of giving breast milk in the last 28 days = NA^4^ & Freq. of formula feeding last 28 days = ‘1-3x a day’** | **Record of the same infant at 3 or 6 mo was excluded** |
| --- | --- | --- | --- | --- | --- | --- | --- | --- |
|  |  | *n* available records | | | *n* excluded records | | | |
| **3** | 148 | 145 | 148 | 148 | 3 | 0 | 0 | - |
| **6** | 149 | 147 | 149 | 149 | 2 | 0 | 0 | 4 |
| **9** | 129 | 124 | 129 | 129 | 5 | 0 | 0 | 2 |
| **12** | 125 | 116 | 125 | 125 | 9 | 0 | 0 | 2 |
| **18** | 109 | 0 | 0 | 0 | 0 | 0 | 0 | 3 |
| **24** | 108 | 0 | 0 | 0 | 0 | 0 | 0 | 2 |
| **Total** | 768 | 532 | 551 | 551 | 19 | 0 | 0 | 13 |

^1’^Yes’ or ‘No’

^2^’>3 times a day’ or ‘1 – 3 times a day’ or ‘>3 times a week’ or ‘1 – 3 times a week’ or ‘Never’

^3^>3 times a day’ or ‘1 – 3 times a day’ or ‘1 – 3 times a month’ or ‘>3 times a week’ or ‘1 – 3 times a week’ or ‘Never’

^4^NA = missing value

**South Africa**

| **Age group** | ***n*** | **‘Are you currently breastfeeding?’^1^** | **‘How often do you give breast milk?’^2^** | **‘How often do you give formula?’^3^** | **Freq. of giving breast milk in the last 28 days = ‘never’** | **Freq. of giving breast milk last in the 28 days = NA^4^ & Freq. of formula feeding last 28 days = ‘>3x a day’** | **Freq. of giving breast milk in the last 28 days = NA^4^ & Freq. of formula feeding last 28 days = ‘1-3x a day’** | **Record of the same infant at 3 or 6 mo was excluded** |
| --- | --- | --- | --- | --- | --- | --- | --- | --- |
|  |  | *n* available records | | | *n* excluded records | | | |
| **3** | 199 | 167 | 194 | 187 | 17 | 2 | 0 | - |
| **6** | 175 | 135 | 173 | 171 | 31 | 1 | 1 | 2 |
| **9** | 173 | 139 | 172 | 169 | 31 | 1 | 0 | 4 |
| **12** | 337 | 169 | 229 | 220 | 59 | 0 | 0 | 24 |
| **18** | 101 | 0 | 0 | 0 | 0 | 0 | 0 | 14 |
| **24** | 138 | 0 | 0 | 0 | 0 | 0 | 0 | 19 |
| **Total** | 1123 | 610 | 768 | 747 | 138 | 4 | 1 | 63 |

^1’^Yes’ or ‘No’

^2^’>3 times a day’ or ‘1 – 3 times a day’ or ‘>3 times a week’ or ‘1 – 3 times a week’ or ‘Never’

^3^>3 times a day’ or ‘1 – 3 times a day’ or ‘1 – 3 times a month’ or ‘>3 times a week’ or ‘1 – 3 times a week’ or ‘Never’

^4^NA = missing value

**Sri Lanka**

| **Age group** | **n** | **‘Are you currently breastfeeding?’^1^** | **‘How often do you give breast milk?’^2^** | **‘How often do you give formula?’^3^** | **Freq. of giving breast milk in the last 28 days = ‘never’** | **Freq. of giving breast milk last in the 28 days = NA^4^ & Freq. of formula feeding last 28 days = ‘>3x a day’** | **Freq. of giving breast milk in the last 28 days = NA^4^ & Freq. of formula feeding last 28 days = ‘1-3x a day’** | **Record of the same infant at 3 or 6 mo was excluded** |
| --- | --- | --- | --- | --- | --- | --- | --- | --- |
|  |  | *n* available records | | | *n* excluded records | | | |
| **3** | 137 | 137 | 137 | 137 | 0 | 0 | 0 | - |
| **6** | 119 | 119 | 119 | 119 | 1 | 0 | 0 | 0 |
| **9** | 106 | 106 | 106 | 106 | 2 | 0 | 0 | 0 |
| **12** | 78 | 77 | 77 | 77 | 2 | 0 | 0 | 0 |
| **18** | 51 | 0 | 0 | 0 | 0 | 0 | 0 | 1 |
| **24** | 47 | 0 | 0 | 0 | 0 | 0 | 0 | 1 |
| **Total** | 538 | 439 | 439 | 439 | 5 | 0 | 0 | 2 |

^1’^Yes’ or ‘No’

^2^’>3 times a day’ or ‘1 – 3 times a day’ or ‘>3 times a week’ or ‘1 – 3 times a week’ or ‘Never’

^3^>3 times a day’ or ‘1 – 3 times a day’ or ‘1 – 3 times a month’ or ‘>3 times a week’ or ‘1 – 3 times a week’ or ‘Never’

^4^NA = missing value

**Supplementary Figure 1:** Flowchart of the sample of total body water measurements.

*n* = 3,756

Difference between weight and (fat mass + fat free mass) >0.1 kg (*n* = 202)

Doubt about age at TBW measurement (*n* = 22)

High deuterium enrichment (likely contamination error) (*n* = 1)

Low deuterium enrichment (likely dose error) (*n* = 8)

Not breastfed at the moment of measuring TBW (*n* = 670)

Premature birth (<37 wks) (*n* = 43)

Davies et al. (*n* = 20) (3)

Haisma et al. (*n* = 55) (4, 5)

Jain et al. (*n* = 133) (6)

Multicenter Infant Body Composition Reference Study (*n* = 4,077) (1, 2)

Thorisdottir et al. (*n* = 21) (7)

UmbiBaby (*n* = 282) (8, 9, 10)

UmbiGodisa (*n* = 30) (8, 11, 12)

Unpublished data (India and Zambia) (*n* = 45)

Unpublished data (follow-up of Thorisdottir et al.) (*n* = 39)

**Supplementary Figure 2:** Flowchart of the sample of breast milk and non-milk water intake measurements.

*n* = 130

Haisma et al. (*n* = 11) (4, 5)

Gibson et al. (*n* = 30) (13)

Unpublished data (India) (*n* = 44)

Unpublished data (Zambia) (*n* = 52)

Unacceptable curve fitting errors (>100 mg/kg) (*n* = 2)

Erroneous enrichment data (*n* = 4)

Incomplete available data (*n* = 1)

**Supplementary Figure 3:** Bland-Altman plots per age group 0.5 mo (≤2), 3 mo (>2 – ≤4.5), 6 mo (>4.5 – ≤7.5), 9 mo (>7.5 – ≤10.5), 12 mo (>**10**.5 – ≤16.5), 18 mo (>16.5 – ≤20.5), 24 mo (> 20.5) to examine bias of total body water (TBW) estimated from body weight using the Cambridge equation (TBW = 0.84*weight^0.82^) (TBW_pred_) relative to TBW measured with isotope dilution (TBW_id_).

**Supplementary Figure 4:** Bland-Altman plots for (A) female and (B) male infants to examine bias of total body water (TBW) estimated from body weight using the Cambridge equation (TBW = 0.84*weight^0.82^) (TBW_pred_) relative to TBW measured with isotope dilution (TBW_id_).

**Supplementary Figure 5:** Bland-Altman plots per country (A = Australia; B = Brazil; C = Iceland; D = India; E = Pakistan; F = South Africa; G = Sri Lanka; H = Zambia) to examine bias of total body water (TBW) predicted from body weight using the Cambridge equation (TBW = 0.84*weight^0.82^) (TBW_pred_) relative to TBW measured with isotope dilution (TBW_id_).

**References**

1.	Murphy-Alford AJ, Johnson W, Nyati LH, Santos IS, Hills AP, Ariff S, et al. Body composition reference charts for infants from birth to 24 months: Multicenter Infant Body Composition Reference Study. The American Journal of Clinical Nutrition. 2023;117(6):1262-9. doi: https://doi.org/10.1016/j.ajcnut.2023.02.012.

2.	Kuriyan R, Hills AP, Murphy-Alford A, Padmanabha R, Nyati LH, Byrne NM, et al. Body composition of infants at 6 months of age using a 3-compartment model. European Journal of Clinical Nutrition. 2023.

3.	Davies PS, Wells J, Lucas A. Adjusting milk intake for body size in early infancy. Early Human Development. 1994;36(1):61-7. doi: https://doi.org/10.1016/0378-3782(94)90033-7.

4.	Haisma H, Coward WA, Visser GH, Vonk R, Wells JC, Wright A, Victora CG. Socio-economic and environmental factors influence energy utilization in Brazilian breast-fed infants. The Journal of Nutrition. 2006;136(11):2945-51. doi: https://doi.org/10.1093/jn/136.11.2945.

5.	Haisma H, Wells JCK, Coward WA, Filho DD, Victora CG, Vonk RJ, Wright A, Visser GH. Complementary Feeding with Cow’s Milk Alters Sleeping Metabolic Rate in Breast-Fed Infants. The Journal of Nutrition. 2005;135(8):1889-95. doi: https://doi.org/10.1093/jn/135.8.1889.

6.	Jain V, Kurpad A, Kumar B, Devi S, Sreenivas V, Paul V. Body composition of term healthy Indian newborns. European Journal of Clinical Nutrition. 2016;70(4):488-93. doi: https://doi.org/10.1038/ejcn.2015.152.

7.	Thorisdottir B, Odinsdottir T, Gunnlaugsson G, Eaton S, Fewtrell MS, Vázquez-Vázquez A, Kleinman RE, Thorsdottir I, Wells JC. Metabolizable energy content of breastmilk supports normal growth in exclusively breastfed Icelandic infants to age 6 months. The American Journal of Clinical Nutrition. 2023;118(2):468-75. doi: https://doi.org/10.1016/j.ajcnut.2023.06.005.

8.	Vannevel V, Vogel JP, Pattinson RC, Adanu R, Charantimath U, Goudar SS, et al. Antenatal Doppler screening for fetuses at risk of adverse outcomes: a multicountry cohort study of the prevalence of abnormal resistance index in low-risk pregnant women. BMJ Open. 2022;12(3):e053622. doi: https://doi.org/10.1136/bmjopen-2021-053622.

9.	Nel S, Feucht UD, Mulol H, Wenhold FAM. Association of Prenatal Placental Function with Anthropometry and Body Composition through 2 years of Age in South African Infants: The UmbiBaby Study. The Journal of Nutrition. 2023;153(4):958-69. doi: https://doi.org/10.1016/j.tjnut.2023.02.007.

10.	Feucht U, Mulol H, Vannevel V, Pattinson R. The ability of continuous-wave Doppler ultrasound to detect fetal growth restriction. PLOS ONE. 2021;16(8):e0255960. doi: https://doi.org/10.1371/journal.pone.0255960.

11.	Nyofane M, Hoffman M, Mulol H, Botha T, Vannevel V, Pattinson R, Feucht U. Early Childhood Growth Parameters in South African Children with Exposure to Maternal HIV Infection and Placental Insufficiency. Viruses. 2022;14(12). doi: https://doi.org/10.3390/v14122745.

12.	Mulol H, Nel S, Wenhold FAM, Feucht UD. Exploring Infant Size and Body Composition at 18 Months: An Ambidirectional Peri-Urban South African Cohort Study. Maternal & Child Nutrition. 2024;n/a(n/a):e13780. doi: https://doi.org/10.1111/mcn.13780.

13.	Gibson RS, Rahmannia S, Diana A, Leong C, Haszard JJ, Hampel D, et al. Association of maternal diet, micronutrient status, and milk volume with milk micronutrient concentrations in Indonesian mothers at 2 and 5 months postpartum. The American Journal of Clinical Nutrition. 2020;112(4):1039-50. doi: https://doi.org/10.1093/ajcn/nqaa200.
